# Review of MAC Protocols for Vehicular Ad Hoc Networks †

**DOI:** 10.3390/s20236709

**Published:** 2020-11-24

**Authors:** Mengyuan Ma, Kai Liu, Xiling Luo, Tao Zhang, Feng Liu

**Affiliations:** 1 School of Electronics and Information Engineering, Beihang University, Beijing 100191, China; Mengyuan@buaa.edu.cn (M.M.); liuk@buaa.edu.cn (K.L.); zhtao@buaa.edu.cn (T.Z.); liuf@buaa.edu.cn (F.L.); 2 Hangzhou Innovation Institute, Beihang University, Hangzhou 310051, China

**Keywords:** vehicular ad hoc networks (VANETs), medium access control (MAC), multi-channel, single-channel, distributed MAC, centralized MAC, dynamic access interval

## Abstract

Vehicular ad hoc networks (VANETs) need to support the timely end-to-end transmissions of safety and non-safety messages. Medium access control (MAC) protocols can ensure fair and efficient sharing of channel resources among multiple vehicles for VANETs, which can provide timely packet transmissions and significantly improve road safety. In this paper, we review the standards of some countries for VANETs. Then, we divide the MAC protocols proposed for VANETs into single-channel MAC protocols and multi-channel MAC protocols according to the number of physical occupied spectrum resources. Both are further discussed based on their hierarchical structures, i.e., distributed and centralized structures. General design and optimization mechanisms of these commonly used MAC protocols for VANETs are reviewed. From the viewpoint of 7 aspects, we compare the advantages and disadvantages of these typical MAC protocols. Finally, we discuss the open issues to improve the MAC performance and future work on MAC design for VANETs.

## 1. Introduction

Vehicular communication is one of the most important technologies for the construction of intelligent transportation systems (ITS) and the smart city [1]. In recent years, with the development and deployment of 5G technology, vehicular ad hoc networks (VANETs) have attracted increasing attention, because they can provide timely transmissions of safety messages and efficient information exchanges, which can guarantee road safety.

**Figure 1:**
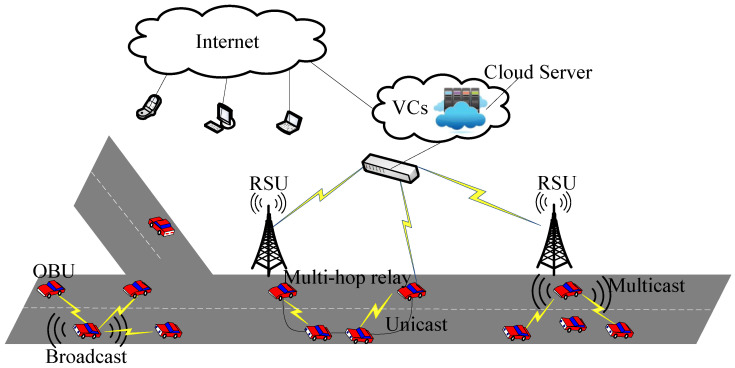
Network structure of VANETs.

As shown in Figure 1, VANET is a special type of mobile ad hoc network (MANET). As mobile intelligent terminals, vehicles are equipped with on-board units (OBUs) to communicate with each other. There are various communication modes among vehicles in VANETs, including single-hop unicast communication, single-hop multicast communication, single-hop broadcast communication and multi-hop relay communication [2]. Medium access control (MAC) protocols can ensure fair channel access among vehicles, reduce access delay, and maximize channel use [3]. With the help of road-side units (RSUs) deployed along the roads, vehicle-to-vehicle (V2V) communication and vehicle-to-infrastructure (V2I) communication are realized [4,5]. Based on virtualization technology, vehicular clouds (VCs) use the information provided by the OBUs and RSUs to form a resource pool, including computing resources, storage resources, network resources, and other resources. The cloud server in the VCs is responsible for resource integration of comprehensive transportation and entertainment information. It can access the Internet like mobile phones, computers, and laptops to form the Internet of Everything (IoE).

Many countries have allocated dedicated frequency bands for vehicles and divided them into different channels for vehicle communication [6]. According to the physical division of spectrum resources, the MAC protocols for VANETs are divided into single-channel MAC protocols and multi-channel MAC protocols. The single-channel MAC protocols mainly solve the resource allocation problem for multiple nodes, by using mechanisms such as carrier sensing [7], time division multiple access (TDMA) [8], space division multiple access (SDMA), and code division multiple access (CDMA). Compared with the single-channel MAC protocols, the multi-channel MAC protocols can simultaneously support packet transmissions on multiple channels and achieve efficient use of channel resources by means of multiple transceivers or channel-switching mechanisms. By load balancing, multi-channel MAC protocols can reduce the possibility of packet collisions of a single channel to a certain extent, which can efficiently improve the network throughput.

For the MAC protocols used in VANETs, it is necessary to have the ability to adapt to the rapid topology changes, high-speed mobility of vehicles, high bandwidth requirements and different quality-of-service (QoS) requirements [9,10,11]. One of the most important goals is to design a good MAC protocol to achieve high throughput for non-safety services while maintaining low latency for road safety applications [12,13]. There are many challenges in designing an efficient MAC protocol for VANETs. The first is the hidden/exposed terminal problems that must be solved in VANETs. Meanwhile, the high-speed moving characteristics of vehicles causes not only drastic topology changes, but also the interruptions of communication links [14,15]. It also faces the problems of insufficient use of a single channel and inefficient load balancing on multiple channels. All of these make channel resource scheduling more challenging in VANETs than other networks [16,17,18]. However, many characteristics and methods, such as the predictable vehicle trajectory, the hierarchical control of network structure, vehicle density-based coordination, and network load conditions, can be used to achieve a better MAC performance for VANETs.

Various MAC protocols have been reviewed for wireless networks. Reference [14] provides a deep discussion on low-power wake-up MAC protocols for wireless sensor network (WSN) from the viewpoints of energy efficiency, latency reduction, and their operation principles. Reference [17] reviews the clustering algorithms in MANETs and gives clustering classification based on mobility, energy, degree, and weight. These reviews provide a basic reference for the MAC protocol design of VANETs. Reference [13] provides a survey on the realizable methods in hybrid MAC protocols for VANETs, which can make use of various combined channel-access schemes to improve the performance of safety and non-safety message delivery. Reference [5] presents discussions on the main characteristics of VANETs, architecture details, constraints of layers, protocols, applications, and future perspectives, which aims to help protocol designers and application engineers to improve the services provided in this type of network. Reference [12] reviews the multi-channel MAC protocols which can dynamically adjust the length of the control channel by using Markov models under different traffic conditions with changing topologies for VANETs. Reference [8] gives more detailed investigation of TDMA-based MAC protocols for VANETs, classifies them into three different categories based on the network topology, identifies the reasons for using the contention-free MAC paradigm in VANETs, and makes comparisons to help readers better understand the difference between the various protocols.

In contrast to previous work, in this paper, we present a more comprehensive introduction to the MAC protocols proposed for VANETs in recent years, compare various MAC protocols, and summarize the innovative mechanisms of existing protocols, such as channel allocation schemes, channel occupancy coordination mechanisms, multi-level architecture management, and timeslot allocation based on traffic environment and/or location characteristics. From more perspectives, we review these related schemes and mechanisms, provide more references for subsequent MAC protocol design for VANETs, and make suggestions for future work.

The main contributions of this paper are as follows:

- The MAC protocols and the typical standards for VANETs are thoroughly reviewed.
- The advantages and disadvantages of the current MAC protocols are discussed based on 7 aspects, including control coordinators, access competition mechanism, communication overhead, multi-channel mechanism, dynamic access interval, time synchronization mechanism, and the number of transceivers.
- The research gaps and potential future work are summarized.

The rest of this paper is organized as follows. Section 2 introduces the related work. The frequency spectrum allocation for VANETs is summarized in Section 3. Section 4 and Section 5 introduce the single-channel and multi-channel MAC protocols respectively, and further divide them into two categories, i.e., distributed and centralized MAC protocols, according to the hierarchical structure. Section 6 compares the related MAC protocols from the viewpoint of 7 aspects. Section 7 concludes this paper with a brief explanation of future work.

## 2. Related Work

The main technologies used in the MAC protocols for safety applications of VANETs are classified into six categories, including multi-channel coordination, clustering, TDMA, SDMA, self-organizing TDMA (STDMA), and cognitive radio [19]. The authors highlight the mechanisms involved and their applicability in future deployment. Reference [20] introduces some multi-channel communication protocols for VANETs before 2013, which points out the challenges unique to the vehicle environment, and discusses synchronization and access for multi-channel coordination. In recent years, many researchers have conducted more in-depth research on the MAC protocols for VANETs, which are divided into distributed MAC protocols and centralized MAC protocols.

Distributed MAC protocols have no central coordinators for access management. Vehicles can use carrier-sense multiple access with collision-avoidance (CSMA/CA) mechanism to complete asynchronous transmissions, or achieve TDMA/SDMA/CDMA-based channel access based on exchanged one-hop neighbor information. In [21,22,23], new schemes are proposed to reduce merging collisions in the distributed TDMA MAC protocols. They assign disjoint sets of timeslots to adjacent areas or lanes, which gives new solutions to decrease packet collisions and improve network throughput. In the adaptive multi-priority distributed multi-channel (APDM) MAC protocol [24], the optimal ratio of control channel interval (CCHI) to service channel interval (SCHI) is derived by using the Markov model, which efficiently use multiple channels. The SDMA mechanism is often used in the distributed MAC protocols. According to the geographic locations of all vehicles, SD-TDMA MAC allocates service channels (SCHs) and timeslots to them. The vehicles in different areas can reuse the same channel according to its predefined SCH and timeslot to realize the application of multiple channels [25]. By adopting a jamming signal, also called black-burst interference, reference [26] varies the access delay for different vehicles during the CCHI and then reduces the access collisions. 

Centralized MAC protocols employ RSUs or cluster heads (CHs) for channel-access management. The RSUs or CHs can provide a powerful access-scheduling function. In the RSU-based centralized MAC protocols, RSUs can easily schedule the channel resources on both single-channel and multiple channels. In the single-channel MAC protocols, an RSU can easily provide the assignment of timeslots, codes, or frequency bands for all vehicles within its coverage, which reduces the overhead of neighbor discovery in a distributed manner. A general way to improve the performance of multi-channel MAC protocols is to adaptively adjust the ratio of CCHI to SCHI in the alternating access channel modes. Reference [27] allows the variable service reservation intervals to reserve SCHs, which makes load balance among control channel (CCH) and SCHs. In reference [28], multi-round elimination on CCH is used to achieve adaptive intervals and the number of rounds is optimized, which can maximize the network throughput. In addition, reference [29] reviews the multi-channel MAC protocols with adaptive variable intervals for VANETs, and points out that it is necessary to dynamically adjust the CCHI and SCHI based on the network conditions and traffic flow conditions. By employing dual transceivers, it is possible to sense safety messages on CCH while transmitting service messages on SCH without missing safety-related messages [30].

The cluster-based MAC protocols present new solutions to channel-access management, in which vehicles in a small area can be grouped into a cluster, and a CH within the cluster is selected to serve as the cluster manager to coordinate channel access and timeslot allocation. The moving CHs reduce the relative motion between vehicles and the possibility of communication link interruption, and extend the lifetime of communication links. In [31], vehicles organize themselves into more stable and non-overlapped clusters by using an adaptive learning mechanism integrated within the fuzzy-logic inference system (FIS) to increase the CH lifetime and the dwell time of its members. By mapping vehicle identification (ID) to channels and timeslots, the disjoint channels and timeslots are assigned to different vehicles to ensure contention-less transmissions [32]. 

**Figure 2:**
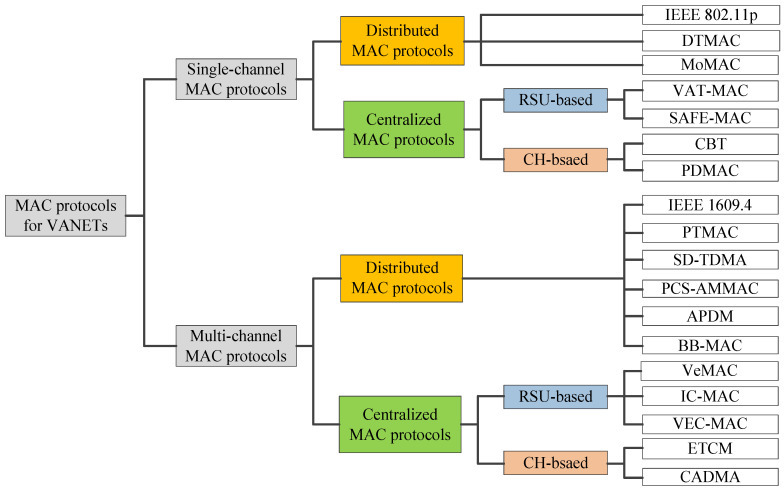
Classification of MAC protocols for VANETs.

Figure 2 summarizes the classification of current MAC protocols for VANETs, which are divided into 4 categories, including distributed single-channel MAC protocols (i.e., IEEE 802.11p, distributed and location-based TDMA MAC (DTMAC) and mobility-aware and collision-avoidance MAC (MoMAC)), centralized single-channel MAC protocols (i.e., adaptive time division multiple access-based MAC (VAT-MAC), speed-aware fairness-enabled MAC (SAFE-MAC), cluster-based TDMA (CBT) and priority-based direction-aware MAC (PDMAC)), distributed multi-channel MAC protocols (i.e., IEEE 1609.4, predictive TDMA MAC (PTMAC), space-division-TDMA (SD-TDMA), priority-based channel-access-asynchronous multi-channel MAC (PCS-AMMAC), adaptive multi-priority distributed multi-channel (APDM) and black-burst-based MAC (BB-MAC)), and centralized multi-channel MAC protocols (i.e., VeMAC, improved coordinated multi-channel MAC (IC-MAC), multi-round elimination contention-based multi-channel MAC (VEC-MAC), enhanced TDMA cluster-based MAC (ETCM), and collision-avoidance directional medium access (CADMA)).

## 3. Spectrum Allocation for VANETs

**Figure 3:**
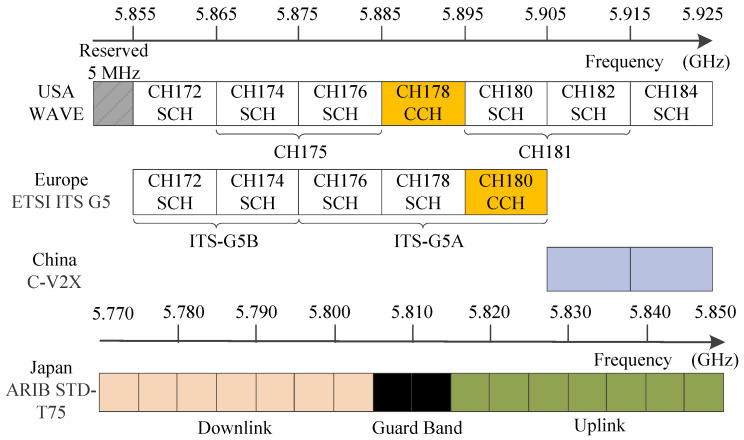
Spectrum allocation for VANETs.

To support the development of vehicular communication, the standard organizations in different countries have assigned dedicated frequency bands for VANETs, and put forward many standards to regulate the communications in VANETs. The allocation of spectrum resources provides the basis for realizing multi-channel operation. Figure 3 shows the frequency spectrum allocations for vehicles in representative countries.

### 3.1. Frequency Band

The Federal Communication Commission (FCC) in the US proposed the wireless access in vehicular environment (WAVE) standard and allocated 75 MHz bandwidth for dedicated short-range communications (DSRC) in 5.9 GHz for V2V and V2I communications [33,34]. In Europe, a total of 50 MHz bandwidth from 5.855 to 5.905 GHz has been allocated by the European Telecommunications Standards Institute (ETSI) to support interoperability among vehicle-mounted equipment and road-side equipment. In Japan, the Association of Radio Industries and Businesses (ARIB) allocated 80 MHz for DSRC as ITS application band in 5.8 GHz, which is used for vehicle information and communication system (VICS) and electronic toll collection (ETC). In addition, the Ministry of Industry and Information Technology (MIIT) of China assigned a dedicated frequency resource of 20 MHz bandwidth in 5.905–5.925 GHz for VANETs.

### 3.2. Multiple Channels

A half-duplex transceiver can sense only a fixed channel, which can be a CCH or a SCH [35]. Multi-channel communications can be realized by switching one half-duplex transceiver to the sensed channels or employing multiple half-duplex transceivers to monitor different channels at the same time. The WAVE standard divides the frequency band into seven 10 MHz channels and reserve 5 MHz band for backup use [36]. One channel serves as the CCH, and is used for exchanging safety-based applications and management information. The rest channels are SCHs for the transmissions of service information (such as entertainment applications). The 10 MHz channel can reduce inter-symbol interference caused by Doppler shift and multipath fading. The standard used in Europe is called ITS-G5 [37]. ETSI divides the frequency band into 5 channels. ITS-G5A uses 3 channels (i.e., 5.875–5.905 GHz) for safety message transmissions and ITS-G5B uses the rest 2 channels (i.e., 5.855–5.875 GHz) for other message transmissions [38]. ARIB STD-T75 divides the frequency band into 14 channels, including 7 uplink channels and 7 downlink channels [39]. 

### 3.3. Modulation

The WAVE standard adopts orthogonal frequency division multiplexing (OFDM) technology [40,41], and all used carriers are orthogonal with each other, which reduces the transmission interference between carriers. It allows data rates from 6 to 27 Mbps. The ITS-G5 standard adopts 2 amplitude-shift keying (2ASK) or 2 phase-shift keying (2PSK) modulation, allowing 6 or 12 Mbps data rate. ARIB STD-T75 uses 2ASK with 1 Mbps data rate support, and quadrature phase-shift keying (QPSK) with 1 or 4 Mbps data rate support [39]. Cellular vehicle to everything (C-V2X) adopted in China is another mainstream technology used in V2X communication [42], including long term evolution-V2X (LTE-V2X) technology and 5G-V2X technology. With 50 Mbps data rate for V2V communication, LTE-V2X technology adopts QPSK, which can reduce the peak-to-average power ratio compared to OFDM, and improve the transmission efficiency of mobile terminals compared to 2ASK. At present, the Internet of Vehicles (IoV) is evolving from LTE-V2X to 5G-V2X in China. 5G-V2X can provide 100 Mbps data rate for V2V communication and 10 Gbps for V2N communication. Data rate is one of the most important performance metrics for VANETs. The higher the data rate, the better the network performance. Table 1 gives the comparison of vehicular communication standards in these countries.

### 3.4. Access Management

The WAVE protocol stack uses IEEE 802.11p as MAC layer access mechanism, and combining with enhanced distributed channel-access (EDCA) mechanism [43,44], divides different types of messages into four different types with increasing priority, i.e., AC [0]–AC [3]. The arbitration inter-frame space number (AIFSN) and contention-window (CW) value are set for different categories, which meets the different QoS requirements. The ITS-G5 standard also employs IEEE 802.11p for access management [45]. It uses decentralized congestion control (DCC) to manage channel congestion by prioritizing data traffic [38]. In the MAC sub-layer, STD-T75 employs adaptive slotted ALOHA for medium access [46]. LTE-V2X defines two complementary communication modes, including LTE-V-Direct and LTE-V-Cell. Through the existing base stations and other equipment, communication can be quickly completed within the network coverage.

## 4. Single-Channel MAC Protocols

Although many national organizations have established dedicated frequency bands to support communication between vehicles and assigned multiple channels, many MAC protocols are limited to the use of a single channel [16]. The single-channel MAC protocols mainly solve the resource allocation and packet collision problems for multiple vehicles on a single channel, by using mechanisms, such as carrier sensing, TDMA, SDMA, and CDMA. According to the hierarchical structure, the single-channel MAC protocols can be further divided into distributed MAC protocols and centralized MAC protocols.

### 4.1. Distributed Single-Channel MAC Protocols

Distributed MAC protocols do not require a central coordinator for access management [47,48]. The research on these protocols mainly focuses on the configuration of contention parameters or improved TDMA access mechanism, which can improve the network performance compared to traditional IEEE 802.11p protocol. In addition, some scholars proposed novel approaches, such as the integration of road characteristics and SDMA technology to achieve timeslot allocation, which makes the access fairer and provide a relatively very low delay.

#### 4.1.1. IEEE 802.11p

IEEE 802.11p is the underlying standard for WAVE, which is based on the back-off competition mechanism [49,50]. It can only transmit safety and non-safety application packets on a single channel, which adopts the priority-based access scheme, integrating the EDCA and CSMA/CA mechanisms. As an asynchronous MAC protocol, IEEE 802.11p allows vehicles to sense the channel status. When the channel is busy or colliding, each access vehicle adopts the mode of random back-off until the channel is idle for a period.

IEEE 802.11p does not provide reliable broadcast mechanism and limited transmission delay, which cannot guarantee the strict QoS requirements of safety applications, such as low delay and high packet delivery rate (PDR) [51,52]. The safety information is broadcasted on the CCH without require-to-send/clear-to-send (RTS/CTS) handshake and acknowledgment (ACK) mechanisms to indicate whether the reception is successful or not, which increases the collision probability of hidden terminals, especially under high traffic load. Therefore, IEEE 802.11p is not the best way for access management in VANETs.

#### 4.1.2. DTMAC

Distributed and location-based TDMA MAC (DTMAC) is designed by taking advantage of the fact that the network topology of vehicles is limited by linear roads in highway scenarios [21]. It can provide reliable broadcast service with limited access delay, and reduce access collision and merging collision problems effectively. Merging collisions refer to the collisions that occur when two vehicles within two-hops that are allocated the same timeslot move into one-hop and still transmit their data packets in their allocated timeslot. Access collisions refer to the collisions that occur when two vehicles compete the same timeslot on the same channel for packet transmissions.

**Figure 4:**
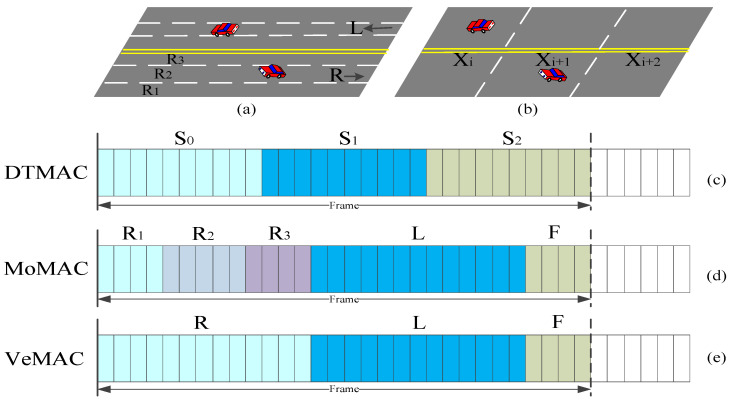
Time structure comparison of DTMAC, MoMAC, and VeMAC.

DTMAC divides the timeslots in each TDMA frame into three parts, which are corresponding to three consecutive areas. The timeslots can be reused outside these three consecutive areas, and the vehicles within 3 adjacent areas can access different timeslots on the channel at the same time without any interference. As shown in Figure 4, the timeslots in each TDMA frame are divided into three parts: S_0_, S_1_, and S_2_, which are respectively occupied by the vehicles in the three continuous areas x_i_, x_i+1_ and x_i+2_.

Because of pre-assigned timeslot sets, the access timeslots of vehicles in adjacent areas are different. Therefore, DTMAC can resolve the hidden terminal and exposed terminal problems, reduce the probability of merging collisions in the same direction, and maximize the use of CCHs [53]. From simulation results, DTMAC achieves a considerably smaller probability of access collisions than VeMAC. However, it requires that each vehicle record the user of each timeslot, the timeslot status (i.e., idle, busy, or in collision), and the type of transmitted packet (i.e., periodic messages or event-driven safety messages) in its additional transmitted frame information (FI) in every packet, to ensure its 2-hop neighboring awareness, which increases communication overhead.

#### 4.1.3. MoMAC

TDMA-based mobility-aware and collision-avoidance MAC (MoMAC) [22] is an extended version of VeMAC [23], which is introduced in Section 5.2.1. It can allocate timeslots to avoid transmission conflicts according to the underlying road topology and lane distribution.

On the multi-lane road, each frame is divided into three groups of time sets, namely L, R, and F. Set F is assigned to RSU for accessing, set L and set R are the timeslots related to the lanes in the left and right directions, respectively. As shown in Figure 4, disjoint timeslots are allocated according to different lanes. Vehicles in the three right lanes occupy different timeslot subsets of R, i.e., R_1_, R_2_, and R3, thus reducing the merging collision problems that may occur in case of vehicles overtaking in the same direction.

By updating the slot occupation information of two-hop neighbors of a vehicle, which is obtained indirectly from its one-hop neighbor, it can detect collisions in some slots and access idle slots in a completely distributed manner. To achieve consistent use of timeslots among adjacent vehicles, each vehicle can choose a free timeslot according to the timeslot occupation information received from adjacent vehicles. Simulation results show that compared with state-of-the-art TDMA MAC protocols, the transmission collisions of MoMAC can be reduced, and the successful transmission rate of safety messages can be greatly enhanced. However, in practical applications, due to inaccurate positioning and temporary loss of global positioning system (GPS) signal, the vehicles may sometimes obtain inaccurate lane information or miss lane change detection, which decreases the MAC performance. Meanwhile, MoMAC cannot handle the unbalanced vehicle density problem in different lanes, which should be resolved by the adaptive timeslot allocation mechanism to match lanes with different vehicle densities.

Figure 4 shows the comparison of slot allocation among DTMAC, MoMAC, and VeMAC.

### 4.2. Centralized Single-Channel MAC Protocols

Centralized MAC protocols have a central coordinator responsible for channel-access management [54]. This coordinator can be a CH or an RSU [55]. When there is such a role for management, the time synchronization problem can be well solved, and it is easy to schedule timeslots without collisions.

RSU can adaptively manage timeslot reservation. However, if the adjacent RSUs reuse the same frequency band, the broadcasting in one region may collide packet transmission in the other neighboring regions [56]. Also, the number of vehicles in a region may vary significantly at any given time, which results in unbalanced network loads and uneven timeslot occupation.

To overcome the challenge caused by high-speed mobility of vehicles, and decrease the high cost of deploying a large number of RSUs, mobile CHs can act as coordinators in the clusters, which can facilitate access management.

#### 4.2.1. VAT-MAC

In adaptive time division multiple access-based MAC (VAT-MAC) protocol, RSU is used to provide access management that can help to improve the network throughput [57].

In VAT-MAC, the RSU broadcasts a time management frame (TMF), including the durations of free transmission period (FTP) and contention period (CP), during the time management period (TMP). FTP is used for packet transmissions of timeslot-allocated vehicles, and unidentified vehicles can compete an idle slot in the CP. If the vehicle successfully accesses a slot in the CP without a collision, the RSU can identify the slot and assign it in the upcoming FTP. Moreover, the RSU knows the collision probability, and based on it, uses the maximum likelihood estimation method to calculate the average vehicle density, so that it can predict the number of newly entering vehicles. Therefore, VAT-MAC can accurately and adaptively optimize each frame length to ensure the efficient use of timeslots and improve network scalability. From the viewpoints of mathematical analysis and simulation experiment, VAT-MAC can significantly improve system scalability and throughput [57].

#### 4.2.2. CBT

In cluster-based TDMA (CBT), the moving CH groups vehicles within its one-hop range and manages their channel access [58]. Every vehicle has its own timeslot for its packet transmission.

**Figure 5:**
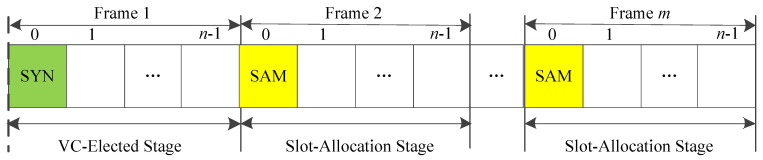
Time structure of CBT.

As shown in Figure 5, the synchronize sequence numbers (SYN) timeslot of the first frame is used to exchange an 8-byte beacon message between adjacent vehicles to mark the start of the frame. The remaining *n* timeslots are used to select a VANET coordinator (VC) as the CH. All VANET nodes (VNs) send a compete-for-VC (CFV) message to compete with each other to win the qualification to be the VC. Once a VN successfully becomes the VC, it periodically transmits beacon signals in the slot 0 of each TDMA frame. In addition, the slot-allocation map (SAM) is used to allocate the slot by the VC.

**Figure 6:**
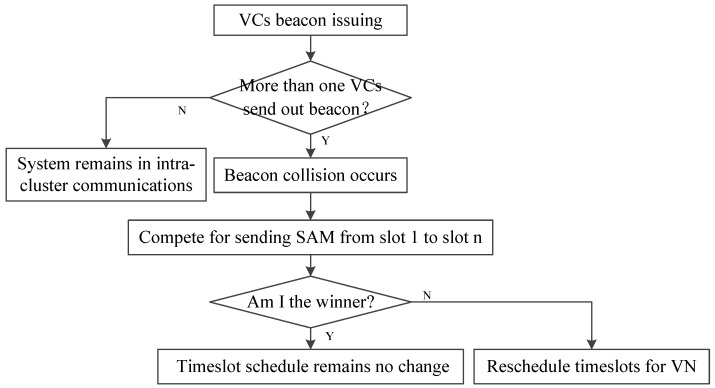
State transitions of inter-cluster communications in CBT.

In contrast to previous protocols which uses election metrics (i.e., speed, direction, relative distance or network connectivity) to choose a CH, CBT uses the above simple transmission monitoring mechanism to select a VC. In Figure 6, when two clusters are closer, a collision may occur, then the two VCs exchange their SAM with each other, the one successfully transmits SAM become the new VC to schedule the timeslot within its communication range. Therefore, it can reduce the average number of timeslots for electing a VC and decrease the time of cluster fusion. Both the analytical and simulation results show that it spends less time to form a small-sized cluster for CBT than IEEE 802.11p.

#### 4.2.3. SAFE-MAC

Speed-aware fairness-enabled MAC (SAFE-MAC) is different from the traditional IEEE 802.11p, which uses CSMA/CA mechanism to access the channel with dynamic MAC parameters, such as the minimum CW, maximum back-off stage, and retransmission limit [59]. It uses mobility metrics of vehicles, including location, direction, and speed, to calculate their residence time in the service area. In addition, the vehicles are divided into 3 batches, according to the duration of stay. Each batch has unique MAC parameters. Then, these parameter values are dynamically changed to ensure that the vehicles with higher speed will be afforded some minimum number of messages, which can guarantee fairness of channel access. However, SAFE-MAC does not consider the fairness issue in case of V2V and V2I communications.

#### 4.2.4. PDMAC

**Figure 7:**
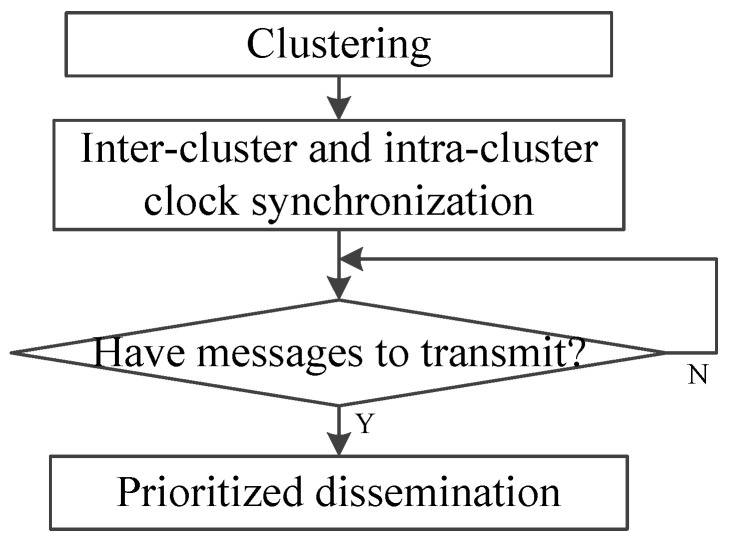
The overview of PDMAC.

Priority-based direction-aware MAC (PDMAC), a cluster-based MAC protocol, emphasizes the low clock synchronization efficiency of the traditional TDMA-based protocol [60]. Figure 7 shows the overview of the PDMAC protocol. After clustering, PDMAC perform inter-cluster and intra-cluster clock synchronization. If a vehicle has messages to transmit, it performs prioritized dissemination.

A new technique of clock synchronization between clusters is introduced by adding the node timer validation bit in the message header. Timer validation bit indicates the clock synchronization status, 1 represents synchronized status, which means that the clock of each vehicle is synchronized with the commonly shared clock of the randomly chosen CH through inter-cluster and intra-cluster clock synchronization. In addition, 0 represents non-synchronized and invalid status. When a vehicle joins the highway, clustering is performed. If the timer validation bit of a vehicle is 0, the clock is required to be synchronized and changed to 1 to realize synchronization of all nodes on the network.

In addition, a three-tier priority assignment method is proposed, including direction-based relay selection, priority based on message type and priority on the basis of severity levels. The first tier of PDMAC selects a relay vehicle with the closest distance to destination vehicle and moving towards it to reduce the packet loss rate. The second tier is used to differentiate the priorities between warning and non-warning messages. The third tier is to determine the priorities of warning messages based on the severity of a critical event. By using the three-tier priority assignment method, PDMAC can send the time-critical warning messages prior to non-warning messages. Simulation and analytical results reveal that compared with other MAC protocols, it can ensure reliable and timely transmission of time-critical messages, and guarantee QoS requirements.

## 5. Multi-Channel MAC Protocols

Generally, the single-radio transceiver is used for vehicular communication. Although dual-radio communication can theoretically double the spectrum efficiency, simultaneous operation on multiple channels may result in increased adjacent channel interference while poorly exploiting the allocated frequency spectrum [20]. When a vehicle is equipped with one half-duplex transceiver, channel switching is adopted to realize multi-channel mechanism. When two half-duplex transceivers are installed on each vehicle, one transceiver always works on the CCH, and the other can turn to any SCH as needed [61]. If so, this guarantees that all vehicles can always receive safety messages from others.

The multi-channel MAC protocols provide various new solutions for improving channel use and reducing latency. The vehicles can use CSMA/CA mechanism to complete asynchronous multi-channel transmission or use TDMA mechanism for synchronous transmission. Compared with the single-channel MAC protocols, the multi-channel MAC protocols can simultaneously support packet transmissions on multiple channels and achieve efficient use of channel resources by means of multiple transceivers or channel-switching mechanisms.

### 5.1. Distributed Multi-Channel MAC Protocols

In distributed multi-channel MAC protocols, each vehicle often broadcasts its one-hop neighbor information on the CCH. By exchanging one-hop neighbor information of neighboring vehicles, channel and timeslot occupancy information of 2-hop range vehicles can be obtained. Therefore, after monitoring a frame or a time interval, a TDMA-based mechanism or a random-access mechanism can be attempted to use the idle timeslot or access the idle channel.

#### 5.1.1. IEEE 1609.4

The WAVE standard consists of IEEE 1609.4 and IEEE 802.11p, and the former specifies an extension of the MAC sub-layer to coordinate multi-channel operation with a single radio [62,63]. The vehicle safety communications consortium (VSCC) recommends that vital safety applications require a frequency of 10 messages per second with a maximum latency of 100 ms [64]. Therefore, most protocols limit the length of a synchronization interval (SI) to 100 ms. Each SI is divided into two parts for the use of CCH and SCH, i.e., CCHI and SCHI, with a guard interval of 4 μs.

**Figure 8:**
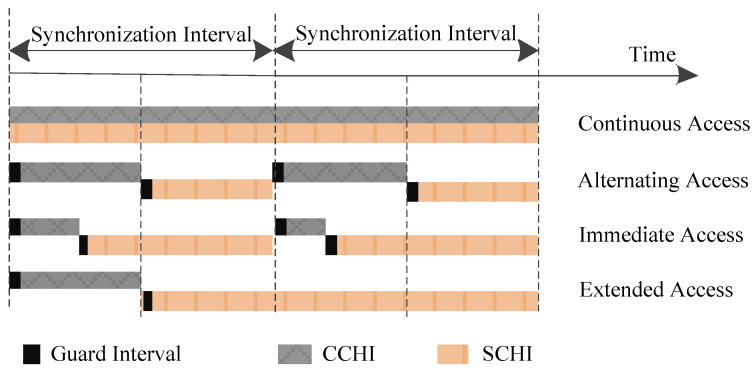
WAVE channel-access operation.

As shown in Figure 8, there are four channel-access modes, i.e., continuous access mode, alternating access mode, immediate access mode and extended access mode [65]. In the continuous access mode, each channel can act as only the CCH or the SCH, and therefore there is no time synchronization problem caused by channel switching. In the alternating access mode, the CCH and SCH are alternately occupied with the equal time interval [66]. The main drawback of this mode is that it limits the use of channel resources under unbalanced control traffic and service traffic. To avoid this problem, immediate access mode allows immediate switching to the SCH to transmit more service messages according to the requirements of channel resources. Extended access mode allows continuous communications over multiple SIs without pauses caused by CCH access which extends the time interval of SCH for transmission.

In the WAVE standard, non-safety applications rely primarily on V2I communication. The RTS/CTS/DATA/acknowledgment (ACK) handshake is performed during the SCHI to complete the transmission of large data packets. The WAVE service advertisement (WSA) sent by the service provider during the CCHI includes the channel number of the SCH to be used. All vehicles within its range decide whether to turn their transceivers to the specific SCH in the upcoming SCHI after receiving the WSA according to their service requirements. However, IEEE 1609.4 does not specify how to select SCH [67]. Moreover, the service provider does not know whether the SCH is selected by the hidden terminals or exposed terminals, and packet transmission conflicts may occur. Therefore, it is important to determine a reasonable SCH allocation mechanism or SCH selection mechanism.

#### 5.1.2. PTMAC

A predictive TDMA MAC (PTMAC) proposes a new collision prediction method that can effectively reduce the number of collisions while maintaining high slot use with very little extra overhead. PTMAC is the first MAC protocol that is applicable to both highway and four-way intersections [68].

**Figure 9:**
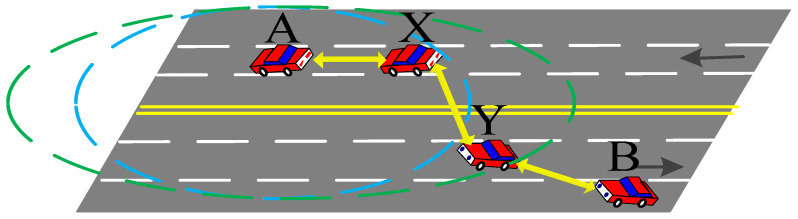
Potential collision detection.

**Figure 10:**
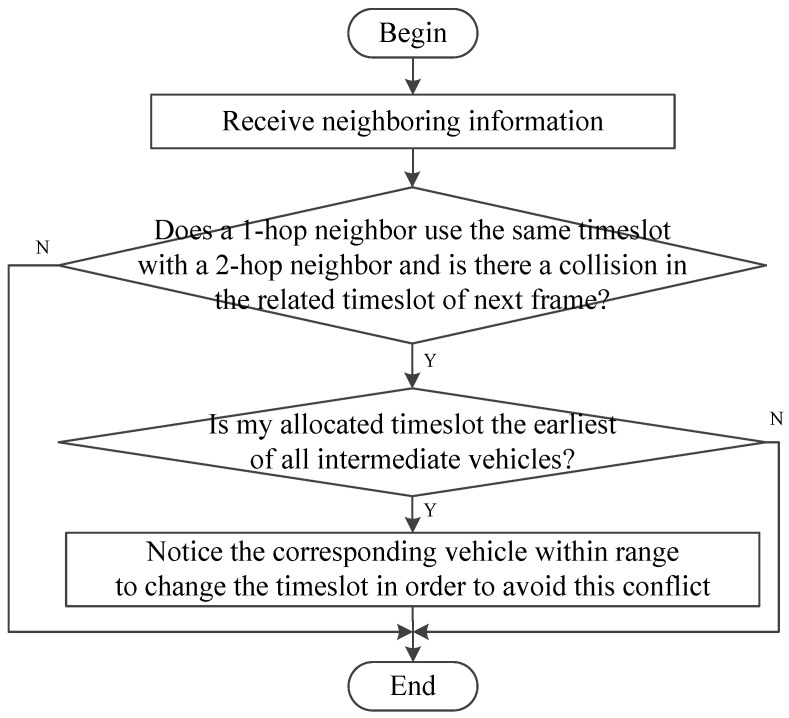
The process of avoiding potential collisions by intermediate vehicles.

The first step of PTMAC is to detect potential conflicts based on slot occupation information. As shown in Figure 9, to avoid the extra cost of broadcasting the information of one-hop and two-hop neighbors, PTMAC uses intermediate vehicles (say X and Y) to detect potential collisions between vehicles outside the two-hop range, say A and B. Then, intermediate vehicles predict whether potential collisions will occur in the future based on real-time traffic conditions and vehicle information. Finally, PTMAC rearranges the timeslots to eliminate this potential conflict. The process of collision prediction and avoidance by intermediate vehicle is shown in Figure 10.

#### 5.1.3. APDM

To minimize and avoid transmission conflicts, it is important to set CCHI and SCHI appropriately. Optimizing and adjusting the length ratio between CCHI and SCHI can achieve the maximum throughput of non-safety applications based on guaranteeing limited delay of reliable safety messages.

APDM is an adaptive MAC protocol with variable interval and without the management of RSUs [24]. It can derive the transmission probabilities of packets with different priorities by using Markov chain and M/M/1 queue, and obtain the optimal ratio *β* of CCHI and SCHI based on the transmission probabilities. Such dynamic interval can improve the channel use because it can maximize service packets throughput on SCHs.

The optimal ratio *β* should satisfy

(1) $$\beta =\frac{T_{CCH}}{T_{SCH}}=\frac{N(1-e^{-\frac{\beta +1}{\beta }\lambda_{e}{\bar{T}}_{ES}\cdot })E[X_{e}]+T_{total}\cdot E[X_{s}]\frac{N_{SCH}}{T_{\mathrm{data}}}}{\left(T_{total}-N(1-e^{-\frac{\beta +1}{\beta }\lambda_{e}{\bar{T}}_{ES}\cdot })E[X_{e}\right]},$$

where *N* is the number of vehicles, *λ*_e_ is the Poisson arrival rate of safety packets, ${\bar{T}}_{ES}$ is the expected time of packet transmissions, *T_total_* is the length of SI, *E*[*X_e_*] is the mean total service time, *E*[*X_s_*] is the average total service time, *N_SCH_* is the number of available SCH, and *T*_data_ is the transmission time of a service packet.

**Figure 11:**
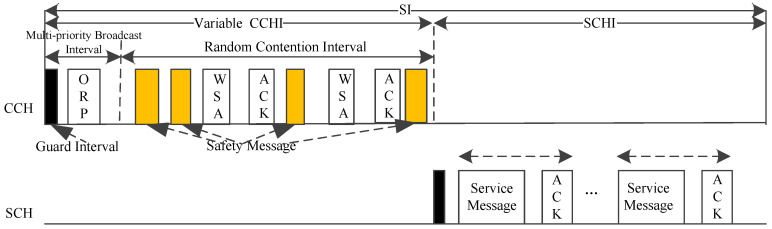
Time structure of APDM.

The vehicle with the smallest MAC address is called the optimizing node (ON). As shown in Figure 11, it broadcasts an optimal ratio packet (ORP) with the best duration of CCHI in multi-priority broadcast interval. During the random contention interval, the vehicles can transmit safety messages and make SCH reservation. An APDM node who has service messages to deliver is a service provider. It reserves the SCH by transmitting WSA packets which contain the service provider MAC address, service information, its channel usage list, and other information. Then the recipients will send an ACK to notify to the service provider. In addition, both the service provider and the recipients tune to the reserved SCH at the beginning of the SCHI for service message transmissions. APDM can work without an RSU by using an ON. It can guarantee the priority transmission of safety packets, reduce the transmission delay, and improve the throughput of the system.

#### 5.1.4. SD-TDMA

**Figure 12:**
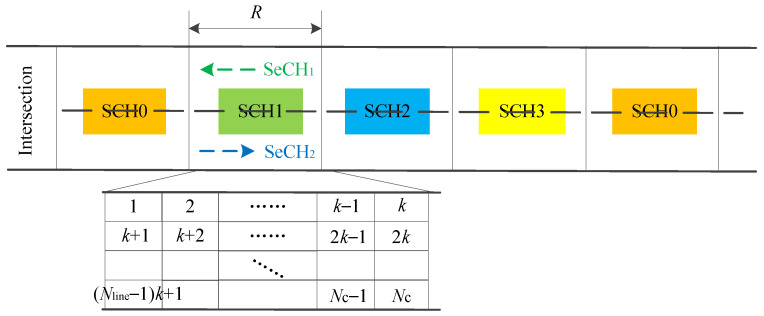
Road segmentation.

**Figure 13:**
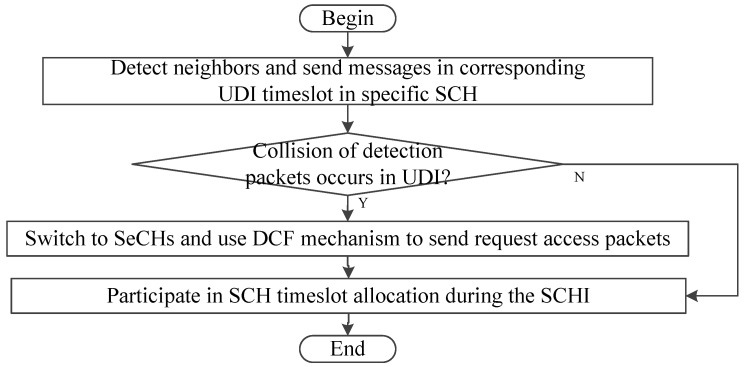
Flowchart of SD-TDMA.

Space-division-TDMA (SD-TDMA) MAC is a multi-channel adaptive MAC protocol [25]. It can dynamically allocate timeslots based on topology changes and reduce transmission collisions. In SD-TDMA, as depicted in Figure 12, four SCHs (i.e., SCH0, SCH1, SCH2 and SCH3) are allocated to the adjacent segments of roads. Two secure channels (SeCHs) are safety channels for two directions of each segment. The CCH is used to transmit event-driven information. The SI is divided into user detection interval (UDI) and SCHI. Vehicles can acquire conflict-free timeslots according to the mapping relationship between timeslots and geographic locations, and access the SCH in UDI to detect neighbors. Once a collision of detection packets occurs in UDI, the vehicles will switch to SeCHs and transmit the request access packets to participate in SCH timeslot allocation during SCHI using distributed coordination function (DCF) mechanism, thus decreasing the collision probability. Figure 13 shows this process.

The vehicles in each segment can use one of the two SeCHs and one of the four SCH simultaneously, and channels are reused along the road. The segment with a length of *R* is divided into *N_c_* cells, and each cell corresponds to a timeslot. Each vehicle can occupy a timeslot based on its location, and then use the dedicated timeslot without collisions, which provides a significantly higher use of timeslots and better fairness for access.

Simulation results show that SD-TDMA can send more beacons than SDMA-based MAC protocols because of eliminating random waiting time before sending. When the vehicle density is low, the neighborhood awareness can be stable at 100%. However, with the increase of vehicle density, the average effective data throughput decreases due to the decrease of SCHI timeslots.

#### 5.1.5. PCS-AMMAC

**Figure 14:**
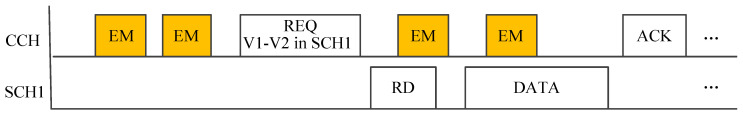
Time structure of PCS-AMMAC.

Priority-based channel-access-asynchronous multi-channel MAC (PCS-AMMAC) proposes an asynchronous multi-channel solution that allows simultaneous transmissions on different SCHs [69]. As shown in Figure 14, the data-negotiation signals and emergency messages (EMs) are transmitted on CCH, the ready (RD) signal and service data are transmitted on SCH.

The EDCA mechanism is used in the data-negotiation phase. The access priority of each vehicle is calculated according to the velocity, location, and traffic and network conditions, and different priorities are mapped to 9 values of the AIFSN, which can provide a fair chance for vehicles to access the channels. When a vehicle wants to send service data, it transmits the data-negotiation message at first, with the details of selected SCH and estimated transmission time of service data. After receiving the ready (RD) signal from receiver side, the sender begins to transmit service data on the specified SCH. Then the recipient replies with an ACK packet on the CCH to notify all nodes to update their local information tables.

PCS-AMMAC can occupy a comparatively free SCH which has lower load depending on the channel usage conditions. In addition, it can use the SCHs in parallel which alleviates the competition probability of multiple vehicles for the SCH and ensures normal work in the scenarios with high service traffic requirements.

#### 5.1.6. BB-MAC

**Figure 15:**
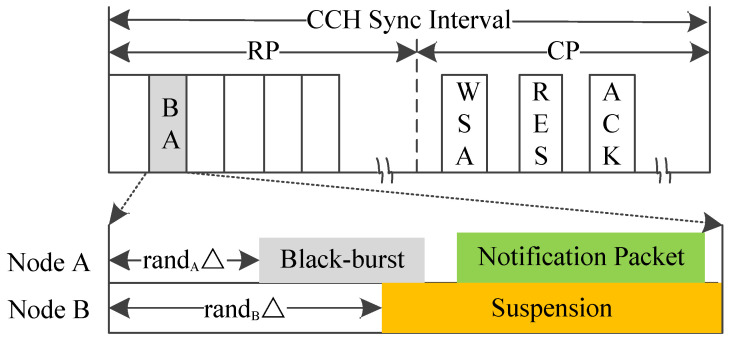
Time structure of BB-MAC.

Black-burst-based MAC (BB-MAC) adopts a timeslot acquisition scheme [26]. As shown in Figure 15, the length of the reservation period (RP) can be adjusted dynamically according to the density of vehicles. During the competition period (CP), vehicles negotiate the use of SCHs by the handshake of WSA/RES/ACK packets. In contrast to traditional TDMA-based slot reservation schemes, in BB-MAC, each vehicle uses the redundancy time at the beginning of some idle timeslot by sending a black-burst after a random delay. By employing black-burst transmission, all access vehicles can detect whether there are other vehicles reserving the current timeslot before them. If there are no collisions in transmitting the black-burst, one of the accessed vehicles successfully reserves the selected timeslot, and reuses the timeslot in the subsequent frames without the black-burst part. Once a vehicle detects the black-burst sent by other vehicles before its black-burst sending time, it suspends its expected transmissions of black-burst and re-selects another idle timeslot for reservation.

Based on the above, BB-MAC can reduce access collisions in the process of reserving timeslots, and speed up timeslot acquisition. Moreover, in BB-MAC, the accessed vehicle which occupies the last timeslot among 2-hop neighbors can switch to the first idle timeslot of the RP to realize dynamic interval adjustment. Therefore, it can adapt to the change of vehicle density. Simulation results also show that BB-MAC has high timeslot acquisition performance.

### 5.2. Centralized Multi-Channel MAC Protocols

Due to the high-speed movement of vehicles, the existence time of vehicles within a range of an RSU is very short, which causes frequent communication interruption [70]. Therefore, the centralized multi-channel MAC protocols should ensure the continuous communication of vehicles and their transmission quality in the presence of dynamic topology changes.

#### 5.2.1. VeMAC

VeMAC is a contention-less TDMA MAC protocol which is suitable for VANETs and can provide efficient one-hop and multi-hop broadcast services on the CCH [23].

**Figure 16:**
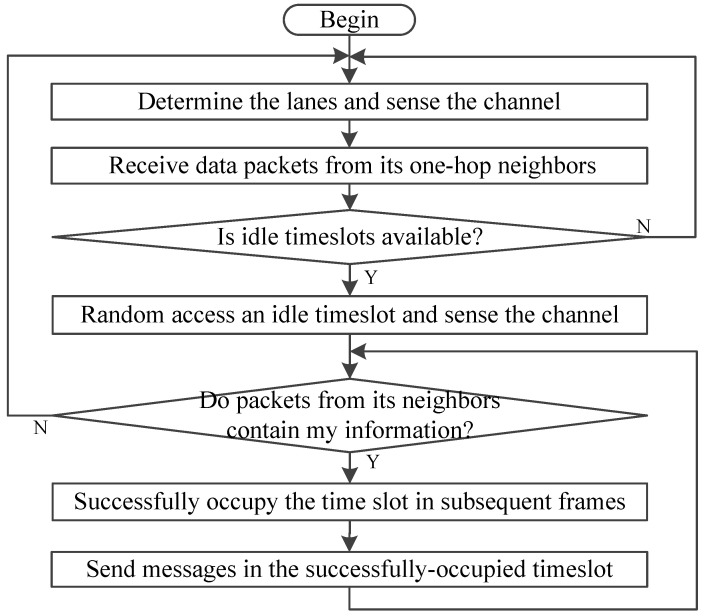
The flowchart of VeMAC.

Vehicles obtain the CCH timeslots in a distributed way, and obtain the SCH timeslots in a centralized way. As shown in Figure 4, VeMAC assigns disjoint time sets (L, R, F) to left- and right-moving vehicles and RSUs on the road. This method reduces the possibility of merging collisions in the opposite direction. As shown in Figure 16, after decoding the data packets from its one-hop neighbors, the vehicles can know the timeslot allocation of the vehicles within its two-hop range and the accessible timeslot of the group. When a vehicle acquires a timeslot, it accesses the same slot until a transmission conflict is detected. Therefore, VeMAC can overcome the hidden and exposed terminal problems caused by vehicle movement [71]. However, it cannot adjust the allocation of timeslots according to vehicle density and the different number of two-way traffic flows.

VeMAC can make full use of multiple channels and support broadcast service on CCH and SCHs by using 2 transceivers [72]. Once a vehicle as service provider announces a service advertisement, other vehicles who want to get the service on the specified SCH transmit their next packet with acceptance of services (AcS) field to notify the service provider this case. In addition, one of their transceivers tunes to that SCH to receive service packets and the other still monitors the CCH. It is shown that VeMAC protocol can provide significantly higher throughput than ADHOC MAC.

#### 5.2.2. IC-MAC

Improved coordinated multi-channel MAC (IC-MAC) improves the use of the SCHs based on C-MAC [49]. As a centralized MAC protocol, RSU is responsible for access management and time synchronization [27]. In contrast to the other MAC protocols using alternating access mode which has the efficiency limitation in high dynamic traffic conditions [73], it adopts dynamic interval schemes and allows SCHI to exceed the half of SI (i.e., 50 ms).

**Figure 17:**
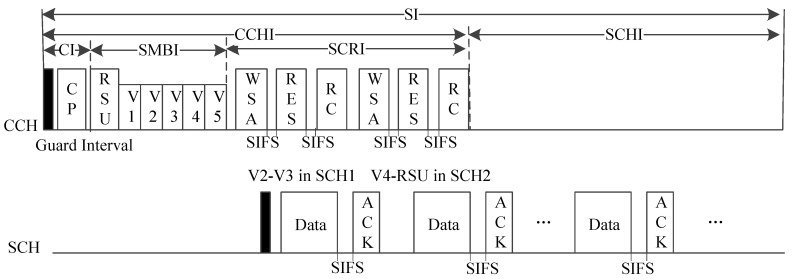
Time structure of IC-MAC.

As shown in Figure 17, when the SI begins, each RSU broadcasts a control packet (CP) during the control interval (CI), including the transmission order of vehicles, and the lengths of the CCHI, safety message broadcast interval (SMBI), and SCHI. Vehicles and RSU broadcast their own safety messages in a TDMA manner during SMBI. Service channel reservation interval (SCRI) is used for SCH reservation; if a vehicle has service messages to transmit, it broadcasts a WSA packet to compete with the channel resources, like the handshake in WAVE. Once a response (RES) which contains the ID of selected SCH and allocated timeslots from the destination vehicle is successfully received, the RSU broadcasts an RSU coordinated (RC) packet within its coverage, including the occupied timeslot and SCHs. After that, the vehicles with successful reservation in the SCRI tune to the specified SCH to transmit their service messages.

In IC-MAC, vehicles can transmit service packets on the selected SCH during the current SCHI or next SCRI after successful reservation. Therefore, the use of SCHs and the throughput of service messages are improved. Simulation results also validate that the delay performance can be improved in high-density scenarios.

#### 5.2.3. VEC-MAC

**Figure 18:**
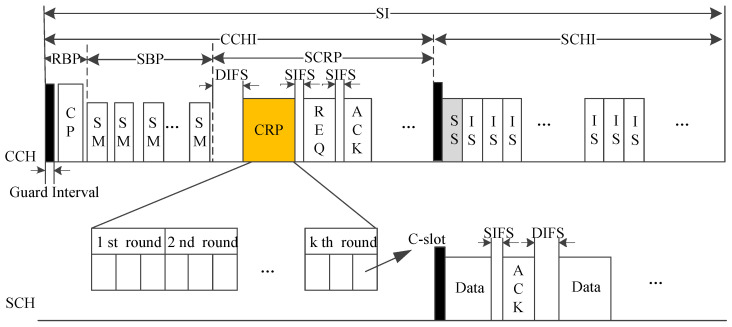
Time structure of VEC-MAC.

In multi-round elimination contention-based multi-channel MAC (VEC-MAC), the RSUs are employed for centralized scheduling [28]. As shown in Figure 18, during the RSU broadcast phase (RBP), the RSU broadcasts a coordination packet (CP), which includes the timeslot scheduling in the safety message broadcast phase (SBP), optimal rounds, and the length of SCH reservation phase (SCRP). Herein, SIFS means short inter-frame space and DIFS means distributed inter-frame space, which are defined in WAVE. Each vehicle can have a timeslot for safety message broadcast (SB) without contention, and broadcast a packet in SCRP to reserve the SCH. The multi-round elimination competition scheme is adopted in the contention resolution period (CRP). The CRP is composed of several contention rounds, and each round includes several contention slots called C-slots. The vehicles randomly choose a C-slot of the first round and attempt to occupy the channel by sending a short energy pulse at the beginning of the C-slot. Then it will perform carrier sensing. If it hears nothing, it enters next round. After it passes the last round, it will become the winner. Then it broadcasts a request (REQ) packet with the information of the destination vehicle and its desired SCH. Once the destination vehicle receives the REQ packet successfully, it will transmit an ACK packet to the winner. After that, they complete data/ACK exchange on the specific SCH.

During the SCHI of the CCH, a newly added vehicle completes its ID recognition process with the RSU by the dynamic slotted ALOHA mechanism. The number of the identification slots (ISs) in the next frame can be estimated as

(2) $$N_{EST}=\frac{\log P_{idle}}{\log(1-(1/N_{cur}))},$$

where *P_idle_* is the ratio of the idle slots to the total ISs in the current frame and *N_cur_* is the number of ISs in the current frame.

As mentioned above, the RSUs can reasonably determine the length of each frame and adaptively adjust the length of CCHI and the number of rounds, and all vehicles can fully use the SCHI on the basis of enough ISs, which leads to system throughput improvement.

#### 5.2.4. ETCM

**Figure 19:**
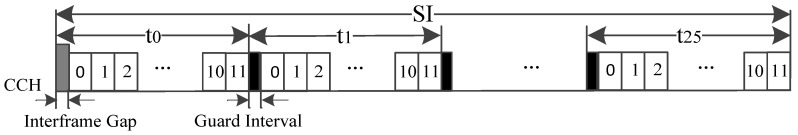
Operation of ETCM protocol.

As an extension of TDMA cluster-based MAC (TC-MAC) [67], enhanced TDMA cluster-based MAC (ETCM) improves the bandwidth use of SCHs with dynamic reallocation of unused slots [32]. It divides the SI into ⌊*N*_max_/*nSCH*⌋ + 1 timeslots, and each timeslot is further divided into *n*SCH × 2 mini-slots. Where ⌊*N*_max_/*nSCH*⌋ is an integer equal to or smaller than *N*_max_/*nSCH*, *N*_max_ represents the maximum number of vehicles in each cluster, and generally equals to 156, and *n* represents the total number of available SCHs, and typically equals to 6. Therefore, there are 26 timeslots in each SI and 12 mini-slots in each timeslot, as shown in Figure 19.

Each vehicle can map its ID assigned by the CH to its corresponding CCH slots and mini-slots, and obtain two mini-slots in two consecutive CCH slots. The calculation of slot numbers of specific CCH timeslots is expressed as

CCHSlot_1_ = (2 × ⌊*ID*/(2 × *nSCH*)⌋ + 1 − *nSCH*/2)%⌊*N*_max_/*nSCH*⌋, (3)

CCHSlot_2_ = (2 × ⌊(*ID* − *ID*%(2 × *nSCH*) + 2 × *nSCH*−1)/(2 × *nSCH*)⌋ + 1 − *nSCH*/2)%⌊*N*_max_/*nSCH*⌋, (4)

Two mini-slots in each timeslot are always reserved for the new entering vehicles with ID 0. Their slot numbers are calculated as

miniSlot_1_ = (*ID* + *nSCH*)%(2 × *nSCH*), (5)

miniSlot_2_ = *ID*%(2 × *nSCH*). (6)

The channel number of selected SCH can also be derived from the mapping of the ID, and can be expressed as

SCHChannel = *ID*%*nSCH*, (7)

SCHSlot = ⌊*ID*/*nSCH*⌋. (8)

When a vehicle has non-safety messages to transmit, handshake exchange is performed between it and destination vehicle in its mini-slot of CCH, and then it can exchange non-safety messages using either its slot, the slot of destination vehicle, or both their slots.

Based on the above, ETCM ensures reliable delivery of time-critical messages by allocating two dedicated mini-slots, increases fairness among vehicles by balanced timeslot allocations, and improves the channel use. Simulation results show that the packet delivery ratio of safety messages is greatly increased to 99% delivery within 50 ms delivery time.

#### 5.2.5. CADMA

Collision-avoidance directional medium access (CADMA) uses a cluster-based polling mechanism [74]. The cluster size is set to the maximum communication distance between two vehicles, allowing direct communication between them. In the polling-based MAC protocols, the control node polls multiple vehicles according to a prearranged schedule.

**Figure 20:**
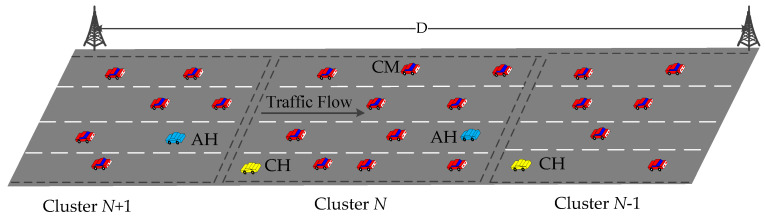
Clustering structure of CADMA.

As shown in Figure 20, the CH is in the back of each cluster, which provides TDMA scheduling to cluster members (CMs) and allocates resources according to QoS threshold. In addition, the protocol sets assist header (AH) as a gateway to help facilitate communication. AH can separate the cluster according to non-response rate and node speed, and maintain the connectivity of clusters. If there is no poll response from a vehicle, it determines that a hole is in the network and works as one-hop relay node controlled by the CH. The presence of AH can solve the network-hole problem when some vehicles are departing from the cluster coverage and they remain in the network-hole between the clusters. Therefore, it can reduce the complexity of cluster combination and separation in VANETs.

In the VANET, safety messages need to be transmitted to the vehicle which is behind the sender to ensure road safety. Therefore, the safety messages must propagate to opposite moving direction of the vehicles. In CADMA, the CH is located at the rear of the cluster, and it controls the transmission direction of the broadcast based on the flow of the vehicle, which can reduce the impact of interference from adjacent clusters, and increase the transmission coverage and spatial reuse. Moreover, the use of directional antennas can support reliable data transmission and higher channel capacity because it is better than omni-directional antenna on connectivity and interference.

## 6. Comparison and Summary

To give more detailed elaboration of each type of MAC protocols, we mainly compare them from 7 aspects, i.e., control coordinators, multi-channel mechanism, access competition mechanism, communication overhead, dynamic access interval, time synchronization mechanism, and the number of transceivers. We aim to find a reasonable trade-off between their pros and cons and then provide reference to the design of MAC protocols. Table 2 and Table 3 compare the distributed MAC protocols and centralized MAC protocols for VANETs, respectively.

- Control Coordinators

Distributed MAC protocols do not require a coordinator. They can better adapt to dynamic topology changes without intervention. Without coordinator management, they use CSMA/CA, TDMA or SDMA mechanism to exchange control packets to acquire useful information and obtain access opportunities through neighbor awareness, which may increase management overhead to some extent. However, due to lack of a coordinator to schedule timeslots, the possibility of contention collisions and merging collisions is high. This collision possibility can be reduced through several measures. For example, DTMAC, MoMAC and SD-TDMA take advantage of the characteristics of road and traffic flow, and use a combination of SDMA and TDMA mechanisms to reduce the possibility of access collisions in a distributed manner by pre-allocating accessible timeslot sets. In addition, in the PTMAC, intermediate vehicles are used to predict and avoid potential collisions.

In centralized MAC protocols, such as VAT-MAC, SAFE-MAC, VeMAC, IC-MAC, and VEC-MAC protocol, the RSUs can provide a powerful access-scheduling function to avoid packet collisions, which increase the network throughput. However, they can only undertake access management within their coverage [75]. When a vehicle moves rapidly, it will frequently enter the coverage of different RSUs, which may cause packet collisions or the vehicles unable to transmit messages. In case of lack of RSUs, local vehicles can select a CH to schedule and manage channel access, which is more suitable for VANETs. The CH can be selected in different ways, as in CBT, PDMAC, ETCM, and CADMA protocols, random competition, and mapping selection metrics with ID and/or network connectivity are adopted. The principle of selecting a local manager is to minimize the impact of communication link interruptions on network stability and communication sessions, and maximize the efficiency of message transmissions. Cooperative communication can solve resource constraint [76]. The cluster-based structure also defines relay vehicles to forward the packets among different clusters, and forms a powerful network architecture for the clusters and for the vehicles within each cluster. A stable CH can reduce the cost of CH reelection, make the structure of the cluster more stable, reduce cluster switching, and facilitate the management of the members in the cluster by the CHs.

- Multi-Channel Mechanism

The multi-channel MAC protocols based on IEEE 802.11p and IEEE 1609.4 standards outperform the single-channel MAC protocols in terms of several key performance indicators, such as the bounded transmission delay of real-time safety applications, the throughput for non-safety applications and adaptability under different traffic densities [8]. It is obvious that the use of a single-channel is inferior to the simultaneous use of multiple channels. This paper mainly discusses the hybrid multi-channel MAC protocols. Therefore, the main problem faced by the protocol design is the reasonable allocation of the transmission interval between the safety messages and service messages (i.e., CCHI and SCHI), and the synchronization problem faced by the channel switching. Implementing dynamic intervals is a common optimization approach to hybrid multi-channel MAC protocols [77,78].

There are also multi-channel MAC protocols that use multiple transceivers to achieve synchronous transmission on CCH and SCHs. Other multi-channel MAC protocols access the mapped service channel by location, ID, or judging the load of the service channel such as ETCM and SD-TDMA MAC. By load balancing, multi-channel MAC protocols can reduce the possibility of collisions of a single-channel to a certain extent, which can efficiently improve throughput of the network.

- Access Competition Mechanism

Contention-based schemes involve carrier sensing mechanisms, e.g., IEEE 802.11p, APDM, and PCS-AMMAC. They work fine in sparse node density. However, they cannot handle dense traffic flow environment. Contention-free MAC protocols access the channel with a defined timeslot or specific code. However, there exist certain access conflicts in the process of competing for the timeslot for the first time and merging collisions in contention-less MAC protocols. Hybrid protocols divide the channel-access time into two periods, i.e., the random-access period for medium access and slot scheduling, and contention-free period for slot use [38]. In recent years, many hybrid MAC protocols have been proposed due to their combination of the advantages of contention-free and contention-based schemes, such as BB-MAC, IC-MAC, and VEC-MAC. Combining with multi-channel mechanism, hybrid MAC protocols show better performance in network throughput, access delay and stability because they cannot only ensure reliable transmission of safety messages through TDMA mechanism, but also guarantee the efficiency of service message delivery through handshake.

- Communication Overhead

In the distributed MAC protocols, vehicles need to exchange the information of timeslot occupancy, code usage, or SCH number about neighboring vehicles, which will increase communication overhead and reduce transmission performance [79,80]. Without RSU management, vehicles access the channel independently, and there is no predefined assignment, which increase the probability of packet collisions to some extent, especially at high traffic load.

The centralized MAC protocols have the scheduling management of the CHs or RSUs, which is beneficial to the arrangement of non-intersecting access channels. Although there are some problems associated with the cost of deploying RSUs, the overhead of the cluster formation and cluster maintenance, and the potential lack of managers, the centralized MAC protocols still have less communication overhead than the distributed MAC protocols.

- Dynamic Access Interval

Single-channel MAC protocols mainly focus on the optimal length of each SI or some period in each SI, and contention-free transmissions on CCH, such as VAT-MAC. In VAT-MAC, RSU is used to predict the number of newly entering vehicles and adaptively adjust frame length through maximum likelihood estimation method, which can maximize CCH use.

Most multi-channel MAC protocols cannot accommodate the dynamic traffic changes. However, some researchers have given a good solution to make full use of SIs by improving the IEEE 1609.4 alternating access mode [81]. A general way to improve the performance of multi-channel MAC protocols is to adaptively adjust the length ratios of CCHI and SCHI in the alternating access mode. For example, BB-MAC estimates the vehicle density based on the collision probability during the access competition, and adjust the interval ratio according to the vehicle density. IC-MAC introduces the SCH reservation and transmission mechanism in the dynamic interval scheme to maximize the SCHI. APDM uses mathematical model to derive the optimal interval ratio for the network, and its dynamic adaptive expansion mechanism allows the lengths of CCHI and SCHI to be optimally adjusted. VEC-MAC can adaptively adjust the length and the number of elimination rounds in the CCHI to improve throughput. These improved dynamic access interval mechanisms for the multi-channel MAC protocols can increase the throughput and packet transmission rate for non-secure applications, and guarantee the QoS of real-time applications.

- Time Synchronization Mechanism

Most MAC protocols for channel access in TDMA mode require a time synchronization mechanism to ensure the starting time of access timeslots or multi-channel-access intervals, such as DTMAC, MoMAC, VAT-MAC, CBT, SAFE-MAC, PDMAC, PTMAC, APDM, SD-TDMA, PCS-AMMAC, BB-MAC, VeMAC, IC-MAC, VEC-MAC, ETCM, and CADMA.

Asynchronous MAC protocols usually use handshake mode for channel-access coordination. They begin to carry out information transmission on a specific channel after obtaining the permission of both vehicles within their coverage. PCS-AMMAC does not require time synchronization. It can switch to a low-load SCH for performance optimization based on network conditions. However, in PCS-AMMAC, the transmissions on the CCH and the SCHs are carried out simultaneously, the vehicles involved in the data transmission may miss the safety packets.

- Number of Transceivers

MAC protocol design and optimization are mostly based on single half-duplex transceiver. Single half-duplex transceiver is enough for the single-channel MAC protocols, such as IEEE 802.11p, DTMAC, MoMAC, VAT-MAC, CBT, SAFE-MAC, and PDMAC. They mainly focus on the CCH use by using optimal back-off mechanism, or adopting TDMA timeslot allocation mechanism based on vehicle density or road characteristics to avoid access collisions and merging collisions.

Multi-channel MAC protocols, such as PTMAC, APDM, SD-TDMA, PCS-AMMAC, BB-MAC, IC-MAC, VEC-MAC, ETCM, and CADMA, mainly focus on the optimal ratio between CCHI and SCHI and choose an appropriate time to switch to SCH for service message transmissions. Vehicles need to negotiate a specific SCH for the exchange of service messages to maximize the utilization of the SCHs.

Multiple transceivers can avoid missing safety messages when handovers between channels, improve efficiency, support simultaneous transmissions on different channels, and ensure that they do not interfere with each other. VeMAC uses 2 transceivers. One transceiver always monitors CCH, and the other can turn to any SCH, which can ensure that the packet transmissions of the CCH and SCHs performed simultaneously. Therefore, event-driven messages can be transmitted at any time and service message transmissions can be completed on any available SCH without multi-channel hidden terminal problems.

Assembling multiple transceivers in automobiles has little effect on automobile manufacturing cost. Therefore, we can consider increasing the number of transceivers when designing MAC layer mechanism to increase channel occupancy. It is noted that using different transceivers for scheduling needs to clarify the frequency band switching mechanism to avoid missing packets.

We also compare the simulation results and conclusions given in the above MAC protocols. It is obvious that the pre-allocation methods of TDMA or SDMA result in less access collisions and merging collisions, although they may cause a waste of channel resources. Statistical results show that their performance is better than that of the traditional CSMA/CA-based IEEE 802.11p protocol, especially at high vehicle density. In addition, the application of multiple channels often brings higher throughput, especially in the adaptive variable access interval mechanisms. Combining with mathematical analysis and prediction, these MAC protocols can get higher performance. The further protocol optimization of priority determination, reasonable channel and timeslot allocation, and dynamic access interval can distinguish the fairness and priority of application messages, and provide QoS guarantee on the basis of efficient channel utilization. Simulation results show that multiple transceivers are worthwhile in exchange for performance improvement.

## 7. Conclusions

This paper has presented a thorough review of MAC protocols designed for VANETs. They are classified into two main categories, i.e., single-channel MAC protocols and multi-channel MAC protocols, which are further divided into distributed MAC protocols and centralized MAC protocols, respectively. Then their typical protocols are described and compared in detail. In the design of VANET MAC protocols, fairness, low latency, high reliability, and high dynamic network topology changes must be considered. However, we found that no current MAC protocol can solve these problems simultaneously.

Based on the comparison, a powerful MAC protocol not only guarantees sufficient fairness, but also needs to meet different QoS requirements, which should distinguish priorities for different services. Safety messages in VANETs should be given the highest priority to ensure their efficient and reliable delivery. Perfect MAC protocols cannot be limited to the use of only single CCH. Multi-channel MAC protocols make full use of multiple channels to provide packet transmissions without interference, which greatly improves the channel utilization. Meanwhile, they should evolve to an adaptive and dynamic access interval mode according to the network load, which leads to high system throughput. In addition, under the premise of unreliable link and high vehicle mobility, the centralized MAC protocols are employed because they can adapt to high dynamic networks and facilitate direct multi-hop forwarding transmissions by using RSUs or CHs. The optimal ratio of intervals, clustering mechanism, multi-channel coordination mechanism and QoS support are some open challenges in the future work. Some new techniques and equipment, such as multiple transceivers and multiple-input multiple-output (MIMO) technique, should also be considered in MAC protocol design to increase throughput.

## Figures and Tables

**Table 1:** Comparison of vehicular communication standards.

	US	Europe	Japan	China
Standard	WAVE	ITS-G5	STD-T75	C-V2X
Frequency band (GHz)	5.9	5.8	5.8	5.9
Frequency range (GHz)	5.850–5.925	5.855–5.905	5.770–5.850	5.905–5.925
Total allocated range (MHz)	75	50	80	20
Number of channels	7 (1 CCH)	5 (1 CCH)	14	1
Data rate (Mbps)	6–27	6, 12	1 or 4	100
Modulation	OFDM	2ASK/2PSK	2ASK/QPSK	QPSK/16QAM/64QAM/256QAM
Number of transceivers	Single	Single	Single	Single

**Table 2:** Comparison of Distributed MAC Protocols.

	DTMAC	MoMAC	PTMAC	APDM	SD-TDMA MAC	PCS-AMMAC	BB-MAC
References	[21]	[22]	[68]	[24]	[25]	[69]	[26]
Published	2016	2018	2016	2017	2018	2017	2019
Coordinator	-	-	-	-	-	-	-
Multi-channel	N	N	Y	Y	Y	Y	Y
Mechanism	Contention-free	Contention-free	Contention-free	Contention-based	Contention-free	Contention-based	Hybrid
Access collision	Not	Not	Solved	Not	Solved	Solved	Solved
Overhead	High	High	High	High	Low	Low	Middle
Dynamic access interval	N	N	N	Y	N	N	Y
Time synchronization	Y	Y	Y	Y	Y	N	Y
Transceivers	1	1	1	1	1	1	1
Simulator	NS2	Sumo	Matlab	NS3	NS2	Matlab	OMNET++

**Table 3:** Comparison of Centralized MAC Protocols.

	VAT-MAC	CBT	SAFE-MAC	PDMAC	VeMAC	IC-MAC	VEC-MAC	ETCM	CADMA
References	[57]	[58]	[59]	[60]	[23]	[27]	[28]	[32]	[74]
Published	2018	2014	2019	2020	2013	2018	2017	2016	2016
Coordinator	RSU	CH	RSU	CH	-	RSU	RSU	CH	CH
Multi-channel	N	N	N	N	Y	Y	Y	Y	Y
Mechanism	Contention-free	Contention-free	Contention-based	Contention-free	Contention-free	Hybrid	Hybrid	Contention-free	Contention-free
Access collision	Not	Solved	Solved	Not	Not	Not	Solved	Not	Not
Overhead	Middle	Low	Low	Low	High	Low	Low	Low	Middle
Dynamic access interval	Y	N	N	N	N	Y	Y	N	N
Time synchronization	Y	Y	Y	Y	Y	Y	Y	Y	Y
Transceivers	1	1	1	1	2	1	1	1	1
Simulator	NS2, Sumo	NS2	-	Matlab	Matlab, NS2	-	Matlab	NS3	Matlab

## References

[1] Maalej Y., Abderrahim A., Guizani M., Hamdaoui B., Balti E. Advanced activity-aware multi-channel operations 1609.4 in vanets for vehicular clouds. Proceedings of the 2016 IEEE Global Communications Conference (GLOBECOM).

[2] Latif S., Mahfooz S., Jan B., Ahmad N., Cao Y., Asif M. (2018). A comparative study of scenario-driven multi-hop broadcast protocols for VANETs. Veh. Commun..

[3] Luo G., Li J., Zhang L., Yuan Q., Liu Z., Yang F. (2018). sdnMAC: A software-defined network inspired MAC protocol for cooperative safety in VANETs. IEEE Trans. Intell. Transp. Syst..

[4] Haider S., Abbas G., Abbas Z.H. VLCS: A Novel Clock Synchronization Technique for TDMA-based MAC Protocols in VANETs. Proceedings of the 2019 4th International Conference on Emerging Trends in Engineering, Sciences and Technology (ICEEST).

[5] Cunha F., Villas L., Boukerche A., Maia G., Viana A., Mini R.A., Loureiro A.A. (2016). Data communication in VANETs: Protocols, applications and challenges. Ad Hoc Netw..

[6] IEEE Computer Society LAN MAN Standards Committee (1997). Wireless LAN Medium Access Control (MAC) and Physical Layer (PHY) Specifications.

[7] Molina-Masegosa R., Gozalvez J., Sepulcre M. (2020). Comparison of IEEE 802.11p and LTE-V2X: An Evaluation with Periodic and Aperiodic Messages of Constant and Variable Size. IEEE Access.

[8] Hadded M., Muhlethaler P., Laouiti A., Zagrouba R., Saidane L.A. (2015). TDMA-based MAC protocols for vehicular ad hoc networks: A survey, qualitative analysis, and open research issues. IEEE Commun. Surv. Tutor..

[9] Ghebleh R. (2018). A comparative classification of information dissemination approaches in vehicular ad hoc networks from distinctive viewpoints: A survey. Comput. Netw..

[10] Hu J., Lyu W., Zhong S., Huang J. (2020). Motion Prediction Based TDMA Protocol in VANETs. Electronics.

[11] Bang J.-H., Lee J.-R. (2020). Collision Avoidance Method Using Vector-Based Mobility Model in TDMA-Based Vehicular Ad Hoc Networks. Appl. Sci..

[12] Nguyen V., Kim O.T.T., Pham C., Oo T.Z., Tran N.H., Hong C.S., Huh E.N. (2018). A survey on adaptive multi-channel MAC protocols in VANETs using Markov models. IEEE Access.

[13] Jayaraj V., Hemanth C., Sangeetha R.G. (2016). A survey on hybrid MAC protocols for vehicular ad-hoc networks. Veh. Commun..

[14] Djiroun F.Z., Djenouri D. (2017). MAC Protocols with Wake-Up Radio for Wireless Sensor Networks: A Review. IEEE Commun. Surv. Tutor..

[15] Huang J., Li Q., Zhong S., Liu L., Zhong P., Wang J., Ye J. (2017). Synthesizing Existing CSMA and TDMA Based MAC Protocols for VANETs. Sensors.

[16] Hadded M., Muhlethaler P., Laouiti A. TDMA scheduling strategies for vehicular ad hoc networks: From a distributed to a centralized approach. Proceedings of the 2018 26th International Conference on Software, Telecommunications and Computer Networks (SoftCOM).

[17] Alinci M., Spaho E., Lala A., Kolici V. Clustering Algorithms in MANETs: A Review. Proceedings of the 2015 Ninth International Conference on Complex, Intelligent, and Software Intensive Systems.

[18] Johari S., Krishna M.B. (2020). TDMA based contention-free MAC protocols for vehicular ad hoc networks: A survey. Veh. Commun..

[19] Gupta N., Prakash A., Tripathi R. (2015). Medium access control protocols for safety applications in vehicular ad-hoc network: A classification and comprehensive survey. Veh. Commun..

[20] Campolo C., Molinaro A. (2013). Multichannel communications in vehicular ad hoc networks: A survey. IEEE Commun. Mag..

[21] Hadded M., Laouiti A., Muhlethaler P., Saidane L.A. An infrastructure-free slot assignment algorithm for reliable broadcast of periodic messages in vehicular ad hoc networks. Proceedings of the 2016 IEEE 84th Vehicular Technology Conference (VTC-Fall).

[22] Lyu F., Zhu H., Zhou H., Qian L., Xu W., Li M., Shen X. (2018). MoMAC: Mobility-aware and collision-avoidance MAC for safety applications in VANETs. IEEE Trans. Veh. Technol..

[23] Omar H.A., Zhuang W., Li L. (2012). VeMAC: A TDMA-based MAC protocol for reliable broadcast in VANETs. IEEE Trans. Mobile Comput..

[24] Song C., Tan G., Yu C., Ding N., Zhang F. (2017). APDM: An adaptive multi-priority distributed multichannel MAC protocol for vehicular ad hoc networks in unsaturated conditions. Comput. Commun..

[25] Xu Z., Wang M., Wu Y., Lin X. (2018). Adaptive multichannel MAC protocol based on SD-TDMA mechanism for the vehicular ad hoc network. IET Commun..

[26] Zhang X., Jiang X., Zhang M. (2018). A black-burst based time slot acquisition scheme for the hybrid TDMA/CSMA multichannel MAC in VANETs. IEEE Wirel. Commun. Lett..

[27] Ma Y., Yang L., Fan P., Fang S., Hu Y. An improved coordinated multichannel MAC scheme by efficient use of idle service channels for VANETs. Proceedings of the 2018 IEEE 87th Vehicular Technology Conference (VTC Spring).

[28] Mao Y., Yan F., Shen L. (2017). Multi-round elimination contention-based multi-channel MAC scheme for vehicular ad hoc networks. IET Commun..

[29] Nguyen V., Pham C., Oo T.Z., Tran N.H., Huh E.N., Hong C.S. (2019). MAC protocols with dynamic interval schemes for VANETs. Veh. Commun..

[30] Song C., Tan G., Yu C. (2017). An Efficient and QoS Supported Multichannel MAC Protocol for Vehicular Ad Hoc Networks. Sensors.

[31] Hafeez K.A., Zhao L., Mark J.W., Shen X., Niu Z. (2013). Distributed Multichannel and Mobility-Aware Cluster-Based MAC Protocol for Vehicular Ad Hoc Networks. IEEE Trans. Veh. Technol..

[32] Shahin N., Kim Y.T. An enhanced TDMA cluster-based MAC (ETCM) for multichannel vehicular networks. Proceedings of the 2016 International Conference on Selected Topics in Mobile & Wireless Networking (MoWNeT).

[33] Shahen Shah A.F.M., Ilhan H., Tureli U. (2019). RECV-MAC: A novel reliable and efficient cooperative MAC protocol for VANETs. IET Commun..

[34] Teixeira F.A., Silva V.F., Leoni J.L., Macedo D.F., Nogueira J.M. (2014). Vehicular networks using the IEEE 802.11 p standard: An experimental analysis. Veh. Commun..

[35] Morgan Y.L. (2010). Notes on DSRC & WAVE standards suite: Its architecture, design, and characteristics. IEEE Commun. Surv. Tutor..

[36] Kim J.-W., Kim J.-W., Jeon D.-K. (2018). A Cooperative Communication Protocol for QoS Provisioning in IEEE 802.11p/Wave Vehicular Networks. Sensors.

[37] Kühlmorgen S., Lu H., Festag A., Kenney J., Gemsheim S., Fettweis G. (2020). Evaluation of Congestion-Enabled Forwarding with Mixed Data Traffic in Vehicular Communications. IEEE Trans. Intell. Transp. Syst..

[38] Haq A.U., Liu K. Review of TDMA-based MAC protocols for vehicular ad hoc networks. Proceedings of the 2018 IEEE 18th International Conference on Communication Technology (ICCT).

[39] Schaffnit T. (2010). Automotive Standardization of Vehicle Networks. Veh. Netw. Automot. Appl. Beyond.

[40] Yao Y., Rao L., Liu X. (2013). Performance and Reliability Analysis of IEEE 802.11p Safety Communication in a Highway Environment. IEEE Trans. Veh. Technol..

[41] Karabulut M.A., Shah A.F.M.S., Ilhan H. (2020). OEC-MAC: A Novel OFDMA Based Efficient Cooperative MAC Protocol for VANETS. IEEE Access.

[42] Nardini G., Virdis A., Campolo C., Molinaro A., Stea G. (2018). Cellular-V2X Communications for Platooning: Design and Evaluation. Sensors.

[43] Gehrsitz T., Kellerer W. (2017). QoS and robustness of priority-based MAC protocols for the in-car power line communication. Veh. Commun..

[44] Liu W., He X., Huang Z., Ji Y. (2019). Transmission Capacity Characterization in VANETs with Enhanced Distributed Channel Access. Electronics.

[45] ETSI TC ITS (2009). European Profile Standard for the Physical and Medium Access Control Layer of Intelligent Transport Systems Operating in the 5 GHz Frequency Band.

[46] Kudoh Y. DSRC standards for multiple applications. Proceedings of the 11th World Congress on ITS.

[47] Li S., Liu Y., Wang J. (2019). An Efficient Broadcast Scheme for Safety-Related Services in Distributed TDMA-Based VANETs. IEEE Commun. Lett..

[48] Thota J., Abdullah N.F., Doufexi A., Armour S. (2020). V2V for Vehicular Safety Applications. IEEE Trans. Intell. Transp. Syst..

[49] Kim Y., Lee M., Lee T.J. (2016). Coordinated multichannel MAC protocol for vehicular ad hoc networks. IEEE Trans. Veh. Technol..

[50] Khan U.A., Lee S.S. (2019). Multi-Layer Problems and Solutions in VANETs: A Review. Electronics.

[51] Abd El-Gawad M.A., Elsharief M., Kim H. (2019). A Comparative Experimental Analysis of Channel Access Protocols in Vehicular Networks. IEEE Access.

[52] Guo W., Huang L., Chen L., Xu H., Xie J. An adaptive collision-free MAC protocol based on TDMA for inter-vehicular communication. Proceedings of the 2012 International Conference on Wireless Communications and Signal Processing (WCSP).

[53] Hadded M., Laouiti A. A Study on Priority-based Centralized TDMA Slot Scheduling Algorithm for Vehicular Ad hoc NETworks. Proceedings of the International Journal of Digital Information and Wireless Communications (IJDIWC).

[54] Cao Y., Zhang H., Zhou X., Yuan D. (2017). A scalable and cooperative MAC protocol for control channel access in VANETs. IEEE Access.

[55] Ma M., Liu K., Zhang T. Review of multi-channel MAC protocols for vehicular ad hoc networks. Proceedings of the 2019 IEEE 19th International Conference on Communication Technology (ICCT).

[56] Zhang R., Cheng X., Yang L., Shen X., Jiao B. (2014). A novel centralized TDMA-based scheduling protocol for vehicular networks. IEEE Trans. Intell. Transp. Syst..

[57] Cao S., Lee V.C. (2017). A novel adaptive TDMA-based MAC protocol for VANETs. IEEE Commun. Lett..

[58] Sheu T.L., Lin Y.H. (2014). A Cluster-Based TDMA System for Inter-Vehicle Communications. J. Inf. Sci. Eng..

[59] Siddik M.A., Moni S.S., Alam M.S., Johnson W.A. (2019). SAFE-MAC: Speed Aware Fairness Enabled MAC Protocol for Vehicular Ad-hoc Networks. Sensors.

[60] Abbas G., Abbas Z.H., Haider S., Baker T., Boudjit S., Muhammad F. (2020). PDMAC: A Priority-Based Enhanced TDMA Protocol for Warning Message Dissemination in VANETs. Sensors.

[61] Boban M., Festag A. (2016). Service-actuated multi-channel operation for vehicular communications. Comput. Commun..

[62] Cao S., Lee V.C.S. (2019). A Novel Coordinated Medium Access Control Scheme for Vehicular Ad Hoc Networks in Multichannel Environment. IEEE Access.

[63] IEEE 1609 Working Group (2016). IEEE Standard for Wireless Access in Vehicular Environments (WAVE)—Multi-Channel Operation.

[64] Hirai T., Murase T. (2020). Performance Evaluations of PC5-based Cellular-V2X Mode 4 for Feasibility Analysis of Driver Assistance Systems with Crash Warning. Sensors.

[65] Boulila N., Hadded M., Laouiti A., Saidane L.A. QCH-MAC: A QoS-aware centralized hybrid MAC protocol for vehicular ad hoc networks. Proceedings of the 2018 IEEE 32nd International Conference on Advanced Information Networking and Applications (AINA).

[66] Nguyen V., Oo T.Z., Chuan P., Hong C.S. (2016). An Efficient Time Slot Acquisition on the Hybrid TDMA/CSMA Multichannel MAC in VANETs. IEEE Commun. Lett..

[67] Almalag M.S., Olariu S., Weigle M.C. TDMA cluster-based MAC for VANETs (TC-MAC). Proceedings of the 2012 IEEE International Symposium on a World of Wireless, Mobile and Multimedia Networks (WoWMoM).

[68] Jiang X., Du D.H. (2016). PTMAC: A prediction-based TDMA MAC protocol for reducing packet collisions in VANET. IEEE Trans. Veh. Technol..

[69] Tripti C., Manoj R. (2017). An asynchronous multi-channel MAC for improving channel utilization in VANET. Procedia Comput. Sci..

[70] Chen C., Liu L., Qiu T., Wu D.O., Ren Z. (2019). Delay-Aware Grid-Based Geographic Routing in Urban VANETs: A Backbone Approach. IEEE/ACM Trans. Net..

[71] Lin Z., Tang Y. (2019). Distributed Multi-Channel MAC Protocol for VANET: An Adaptive Frame Structure Scheme. IEEE Access.

[72] Omar H.A., Zhuang W., Abdrabou A., Li L. (2013). Performance evaluation of VeMAC supporting safety applications in vehicular networks. IEEE Trans. Emerg. Topics Comput..

[73] Babu S., Patra M., Murthy C.S.R. (2015). A novel context-aware variable interval MAC protocol to enhance event-driven message delivery in IEEE 802.11 p/WAVE vehicular networks. Veh. Commun..

[74] Ji S., Kim J., You C. (2016). CADMA: Collision-avoidance directional medium access for vehicular ad hoc networks. Wirel. Netw..

[75] Nguyen V., Khanh T.T., Oo T.Z., Tran N.H., Huh E., Hong C.S. (2019). A Cooperative and Reliable RSU-Assisted IEEE 802.11p-Based Multi-Channel MAC Protocol for VANETs. IEEE Access.

[76] Liu K., Wang R., Yue C., Liu F., Lu T., Xiong Z. (2019). Interference Range-Reduced Cooperative Multiple Access with Optimal Relay Selection for Large Scale Wireless Networks. Sensors.

[77] Nguyen V., Anh Khoa T., Zin T., Tran N., Seon Hong C., Huh E.-N. (2018). Time Slot Utilization for Efficient Multi-Channel MAC Protocol in VANETs. Sensors.

[78] Singh V.K., Kumar R. (2019). An optimized multichannel MAC scheme with dynamic control channel interval in dense VANET. Int. J. Inf. Tecnol..

[79] Li S., Liu Y., Wang J., Ge Y., Deng L., Deng W. (2019). TCGMAC: A TDMA-based MAC protocol with collision alleviation based on slot declaration and game theory in VANETS. Trans. Emerg. Telecommun. Technol..

[80] Lyu F., Zhu H., Zhou H., Xu W., Zhang N., Li M., Shen X. (2017). SS-MAC: A novel time slot-sharing MAC for safety messages broadcasting in VANETs. IEEE Trans. Veh. Technol..

[81] Almohammedi A.A., Noordin N.K., Sali A., Hashim F., Balfaqih M. (2018). An Adaptive Multi-Channel Assignment and Coordination Scheme for IEEE 802.11p/1609.4 in Vehicular Ad-Hoc Networks. IEEE Access.

